# Clustered Regularly Interspaced Short Palindromic Repeats Genotyping of Multidrug-Resistant *Salmonella* Heidelberg Strains Isolated From the Poultry Production Chain Across Brazil

**DOI:** 10.3389/fmicb.2022.867278

**Published:** 2022-06-17

**Authors:** Daniel F. M. Monte, Matthew A. Nethery, Hanna Berman, Shivaramu Keelara, Nilton Lincopan, Paula J. Fedorka-Cray, Rodolphe Barrangou, Mariza Landgraf

**Affiliations:** ^1^Department of Food and Experimental Nutrition, Faculty of Pharmaceutical Sciences, Food Research Center, University of São Paulo, São Paulo, Brazil; ^2^Laboratory of Avian Pathology, Department of Pathology, Theriogenology, and One Health, São Paulo State University (FCAV-Unesp), São Paulo, Brazil; ^3^Genomic Sciences Graduate Program, North Carolina State University, Raleigh, NC, United States; ^4^Department of Food, Bioprocessing and Nutrition Sciences, North Carolina State University, Raleigh, NC, United States; ^5^Department of Population Health and Pathobiology, College of Veterinary Medicine, North Carolina State University, Raleigh, NC, United States; ^6^Department of Microbiology, Institute of Biomedical Sciences, University of São Paulo, São Paulo, Brazil; ^7^Department of Clinical Analysis, Faculty of Pharmaceutical Sciences, University of São Paulo, São Paulo, Brazil

**Keywords:** antibiotic resistance, CRISPR, phylogeny, *Salmonella* Heidelberg, foodborne disease, WGS

## Abstract

*Salmonella enterica* subsp. *enterica* serovar Heidelberg has been associated with a broad host range, such as poultry, dairy calves, swine, wild birds, environment, and humans. The continuous evolution of *S*. Heidelberg raises a public health concern since there is a global dispersal of lineages harboring a wide resistome and virulome on a global scale. Here, we characterized the resistome, phylogenetic structure and clustered regularly interspaced short palindromic repeats (CRISPR) array composition of 81 *S*. Heidelberg strains isolated from broiler farms (*n* = 16), transport and lairage (*n* = 5), slaughterhouse (*n* = 22), and retail market (*n* = 38) of the poultry production chain in Brazil, between 2015 and 2016 using high-resolution approaches including whole-genome sequencing (WGS) and WGS-derived CRISPR genotyping. More than 91% of the *S*. Heidelberg strains were multidrug-resistant. The total antimicrobial resistance (AMR) gene abundances did not vary significantly across regions and sources suggesting the widespread distribution of antibiotic-resistant strains from farm to market. The highest AMR gene abundance was observed for *fosA7*, *aac(6′)-Iaa*, *sul2*, *tet(A)*, *gyrA*, and *parC* for 100% of the isolates, followed by 88.8% for *bla*_*CMY*–2_. The β-lactam resistance was essentially driven by the presence of the plasmid-mediated AmpC (pAmpC) *bla*_*CMY*–2_ gene, given the isolates which did not carry this gene were susceptible to cefoxitin (FOX). Most *S*. Heidelberg strains were classified within international lineages, which were phylogenetically nested with *Salmonella* strains from European countries; while CRISPR genotyping analysis revealed that the spacer content was overall highly conserved, but distributed into 13 distinct groups. In summary, our findings underscore the potential role of *S*. Heidelberg as a key pathogen disseminated from farm to fork in Brazil and reinforce the importance of CRISPR-based genotyping for salmonellae. Hence, we emphasized the need for continuous mitigation programs to monitor the dissemination of this high-priority pathogen.

## Introduction

*Salmonella enterica* subsp. *enterica* serovar Heidelberg is most often associated with eggs and poultry (Hennessy et al., 2004; Chittick et al., 2006; Foley et al., 2008; Folster et al., 2012; Jackson et al., 2013). However, this scenario has changed since *Salmonella* Heidelberg has been associated with other sources from a broad host range, such as dairy calves (Centers for Disease Control and Prevention [CDC], 2017), swine (Cabral et al., 2017), wild birds (Liakopoulos et al., 2016a), environmental sources (Antony et al., 2018), human-derived clinical specimens (Abdullah et al., 2021), and outbreaks (Antony et al., 2018), which denotes their importance as a high-priority pathogen.

One of the most important risk factors surrounding foodborne illness is the international food trade that has been circumstantially accompanied by Salmonellae dispersal beyond borders. In this context, there is a global dispersal of multidrug-resistant lineages of serovar *S*. Heidelberg, reaching various countries in North America (Andrysiak et al., 2008; Centers for Disease Control and Prevention, 2014; Public Health Agency of Canada, 2014; Deblais et al., 2018; Cox et al., 2021), South America (Kipper et al., 2021), Europe (Liakopoulos et al., 2016b; Campos et al., 2018), and Asia (Wu et al., 2013). Therefore, the simultaneous increase and extended protraction of *S*. Heidelberg in many parts of the world have favored their genetic acquisition of virulence and antimicrobial resistance (AMR) genes through horizontal gene transfer (HGT), which has ultimately led to one of the most pressing global concerns.

Owing to their importance as a key poultry producer globally, Brazil quickly became the hotspot of *S*. Heidelberg and urgent actions were needed from the food safety authorities to mitigate this pathogen in order to reduce the economic losses in the poultry sector. In this context, most investigations to detect *Salmonella enterica* serovars in the poultry sector still had important methodological gaps, since the food industry focuses especially on *Salmonella* Typhimurium and *Salmonella* Enteritidis, which demonstrate the need for a combined approach between classical microbiology and high-resolution methods such as whole-genome sequencing (WGS) and clustered regularly interspaced short palindromic repeats (CRISPR) genotyping (Barrangou and Dudley, 2016; Thompson et al., 2018; Yousfi et al., 2020). Indeed, the use of these high-throughput sequencing analyses exemplifies a useful means, not only for identifying *Salmonella* serovars but also to trace back the origin of the contamination conferring a substantial aid in decision-making to the poultry sector. In this regard, we demonstrated the usefulness of WGS-based identification in our previous study for genotyping rare *Salmonella enterica* serovars isolated from food and related sources (Monte et al., 2021). This previous survey demonstrated that the CRISPR arrays were highly conserved, and this genomic inspection provides high-resolution genotyping of *Salmonella* serovars. Hence, we performed a genomic study by combining WGS and CRISPR genotyping to characterize *S*. Heidelberg isolates from different sources at broiler farms, slaughterhouses, transport, lairages, and retail markets in Brazil.

## Materials and Methods

### *Salmonella* Heidelberg Strains and Antimicrobial Susceptibility Testing

A total of 79 non-duplicate *Salmonella enterica* subsp. *enterica* serovar Heidelberg from our collection that included isolates obtained from broiler farms (*n* = 16), transport and lairage (*n* = 5), slaughterhouses (*n* = 22), and retail markets (*n* = 38) in Brazil between 2015 and 2016 were used in this study (refer Table 1). We also included two *S*. Heidelberg strains (SH159 and SSc139) from our previous work (Monte et al., 2019) for comparative purposes, totalizing eighty-one isolates. The *Salmonella* isolation was performed according to the International Organization for Standardization (Anonymous, 2007, 2017). These isolates were serotyped on the basis of somatic O, phase 1, and phase 2 of H flagellar antigens by agglutination tests with antisera as specified in the Kauffmann–White–Le Minor scheme (Grimont and Weil, 2007; Guibourdenche et al., 2010).

**TABLE 1:** Features of *S*. Heidelberg strains (*n* = 81) isolated from different sources.

Strain ID	Location*/year	Origin	Source	Resistance profile	Resistance genes	Sequence type
SH018 GCA_002270265.1	SP/2016	Farm	Broiler chicken	FOX-TET-AXO-AUG2-CIP-NAL-XNL-AMP	*bla*_CMY–2_, *fosA7*, *sul2*, *tet(A), aac(6′)-Iaa, gyrA:p.S83F, parC:p.T57S*	ST15
SH019 GCA_002260805.1	SP/2016	Farm	Broiler chicken	FOX-TET-AXO-AUG2-CIP-NAL-XNL-AMP	*bla*_CMY–2_, *fosA7*, *sul2*, *tet(A), aac(6′)-Iaa, gyrA:p.S83F, parC:p.T57S*	ST15
SH10211124 GCA_006332685.1	SC/2016	Farm	Broiler chicken	TET-CIP-NAL	*fosA7*, *sul2*, *tet(A), aac(6′)-Iaa, gyrA:p.S83F, parC:p.T57S*	ST15
SH10227492 GCA_006291695.1	SC/2016	Farm	Broiler chicken	TET-CIP-NAL-STR	*fosA7*, *sul2*, *tet(A), aac(6′)-Iaa, gyrA:p.S83F, parC:p.T57S*	ST15
SH10230633 GCA_004161895.1	MS/2016	Farm	Broiler chicken	FOX-TET-AXO-AUG2-CIP-NAL-XNL-AMP	*bla*_CMY–2_, *fosA7*, *sul2*, *tet(A), aac(6′)-Iaa, aph(3′)-Ia, gyrA:p.S83F, parC:p.T57S*	ST15
SH10190712 GCA_011157915.1	PR/2016	Farm	Broiler chicken	FOX-TET-AXO-AUG2-CIP-NAL-XNL-AMP	*bla*_CMY–2_, *fosA7*, *sul2*, *tet(A), aac(6′)-Iaa, gyrA:p.S83F, parC:p.T57S*	ST15
SH10201911 GCA_011519745.1	SC/2016	Farm	Broiler chicken	TET-CIP-NAL	*fosA7*, *sul2*, *tet(A), aac(6′)-Iaa, gyrA:p.S83F, parC:p.T57S*	ST15
SH10206799 GCA_011520545.1	SC/2016	Farm	Broiler chicken	TET-CIP-NAL	*fosA7*, *sul2*, *tet(A), aac(6′)-Iaa, gyrA:p.S83F, parC:p.T57S*	ST15
SH10225532 GCA_007640935.1	SC/2016	Farm	Broiler chicken	TET-CIP-NAL	*fosA7*, *sul2*, *tet(A), aac(6′)-Iaa, gyrA:p.S83F, parC:p.T57S*	ST15
STy012 GCA_011606045.1	SP/2015	Farm	Broiler chicken	TET-CIP-NAL	*fosA7*, *sul2*, *tet(A), aac(6′)-Iaa, gyrA:p.S83F, parC:p.T57S*	ST15
SI015 GCA_011598585.1	SP/2015	Farm	Broiler chicken	TET-CIP-NAL	*fosA7*, *sul2*, *tet(A), aac(6′)-Iaa, gyrA:p.S83F, parC:p.T57S*	ST15
SH134 GCA_011158435.1	SP/2016	Farm	Chicken cage after cleaning	FOX-TET-AXO-AUG2-CIP-NAL-XNL-AMP	*bla*_CMY–2_, *fosA7*, *sul2*, *tet(A), aac(6′)-Iaa, gyrA:p.S83F, parC:p.T57S*	ST15
SH159 GCA_011157595.1	MG/2016	Farm	Chicken cage after cleaning	FOX-TET-AXO-AUG2-CIP-NAL-XNL-AMP	*bla*_CMY–2_, *fosA7*, *sul2*, *tet(A), aac(6′)-Iaa, gyrA:p.S83F, parC:p.T57S*	ST15
SH415 GCA_006332505.1	SC/2016	Farm	Chicken cage after cleaning	FOX-TET-AXO-AUG2-CIP-NAL-XNL-AMP	*bla*_CMY–2_, *fosA7*, *sul2*, *tet(A), aac(6′)-Iaa, gyrA:p.S83F, parC:p.T57S*	ST15
SH434 GCA_006291935.1	SC/2016	Farm	Chicken cage after cleaning	FOX-TET-AXO-AUG2-CIP-NAL-XNL-AMP	*bla*_CMY–2_, *fosA7*, *sul2*, *tet(A), aac(6′)-Iaa, gyrA:p.S83F, parC:p.T57S*	ST15
SH715 GCA_003874535.1	SP/2016	Farm	Chicken cage after cleaning	TET-CIP-GEN-NAL-STR	*fosA7, aac(3)-VIa, aadA1, sul2, tet(A), aac(6′)-Iaa, gyrA:p.S83F, parC:p.T57S*	ST15
SH264 GCA_010933975.1	PR/2016	Transport and lairage	Truck after cleaning	FOX-TET-AXO-AUG2-CIP-NAL-XNL-AMP	*bla*_CMY–2_, *fosA7*, *sul2*, *tet(A), aac(6′)-Iaa, gyrA:p.S83F, parC:p.T57S*	ST15
SH265 GCA_010884255.1	PR/2016	Transport and lairage	Truck after cleaning	FOX-TET-AXO-AUG2-CIP-NAL-XNL-AMP	*bla*_CMY–2_, *fosA7*, *sul2*, *tet(A), aac(6′)-Iaa, gyrA:p.S83F, parC:p.T57S*	ST15
SH414 GCA_003877275.1	SC/2016	Transport and lairage	Truck after cleaning	FOX-TET-AXO-AUG2-CIP-NAL-XNL-AMP-STR	*bla*_CMY–2_, *fosA7*, *sul2*, *tet(A), aac(6′)-Iaa, gyrA:p.S83F, parC:p.T57S*	ST15
SH433 GCA_006332565.1	SC/2016	Transport and lairage	Truck after cleaning	FOX-TET-AXO-AUG2-CIP-NAL-XNL-AMP	*bla*_CMY–2_, *fosA7*, *sul2*, *tet(A), aac(6′)-Iaa, gyrA:p.S83F, parC:p.T57S*	ST15
SH435 GCA_006291875.1	SC/2016	Transport and lairage	Truck after cleaning	FOX-TET-AXO-AUG2-CIP-NAL-XNL-AMP	*bla*_CMY–2_, *fosA7*, *sul2*, *tet(A), aac(6′)-Iaa, gyrA:p.S83F, parC:p.T57S*	ST15
SH122 GCA_011616265.1	SP/2016	Slaughterhouse	Chicken carcass	FOX-TET-AXO-AUG2-CIP-NAL-XNL-AMP	*bla*_CMY–2_, *fosA7*, *sul2*, *tet(A), aac(6′)-Iaa, gyrA:p.S83F, parC:p.T57S*	ST15
SH125 GCA_011544755.1	SP/2016	Slaughterhouse	Chicken carcass	FOX-TET-AXO-AUG2-CIP-NAL-XNL-AMP-STR	*bla*_CMY–2_, *fosA7*, *sul2*, *tet(A), aac(6′)-Iaa, gyrA:p.S83F, parC:p.T57S*	ST15
SH128 GCA_010956115.1	SP/2016	Slaughterhouse	Chicken carcass	FOX-TET-AXO-AUG2-CIP-NAL-XNL-AMP	*bla*_CMY–2_, *fosA7*, *sul2*, *tet(A), aac(6′)-Iaa, gyrA:p.S83F, parC:p.T57S*	ST15
SH129 GCA_011591705.1	SP/2016	Slaughterhouse	Chicken carcass	FOX-TET-AXO-AUG2-CIP-NAL-XNL-AMP	*bla*_CMY–2_, *fosA7*, *sul2*, *tet(A), aac(6′)-Iaa, gyrA:p.S83F, parC:p.T57S*	ST15
SH258 GCA_011533705.1	PR/2016	Slaughterhouse	Chicken carcass	FOX-TET-AXO-AUG2-CIP-NAL-XNL-AMP	*bla*_CMY–2_, *fosA7*, *sul2*, *tet(A), aac(6′)-Iaa, gyrA:p.S83F, parC:p.T57S*	ST15
SH266 GCA_011157875.1						
GCA_011157875.1	PR/2016	Slaughterhouse	Chicken carcass	FOX-TET-AXO-AUG2-CIP-NAL-XNL-AMP	*bla*_CMY–2_, *fosA7*, *sul2*, *tet(A), aac(6′)-Iaa, gyrA:p.S83F, parC:p.T57S*	ST15
SH283 GCA_011516545.1	SP/2016	Slaughterhouse	Chicken carcass	FOX-TET-AXO-AUG2-CIP-NAL-XNL-AMP	*bla*_CMY–2_, *fosA7*, *sul2*, *tet(A), aac(6′)-Iaa, gyrA:p.S83F, parC:p.T57S*	ST15
SH284 GCA_010005265.1	SP/2016	Slaughterhouse	Chicken carcass	FOX-TET-AXO-AUG2-CIP-NAL-XNL-AMP	*bla*_CMY–2_, *fosA7*, *sul2*, *tet(A), aac(6′)-Iaa, gyrA:p.S83F, parC:p.T57S*	ST15
SH285 GCA_006291795.1	SP/2016	Slaughterhouse	Chicken carcass	FOX-TET-AXO-AUG2-CIP-NAL-XNL-AMP	*bla*_CMY–2_, *fosA7*, *sul2*, *tet(A), aac(6′)-Iaa, gyrA:p.S83F, parC:p.T57S*	ST15
SSc148 GCA_003877035.1	DF/2016	Slaughterhouse	Chicken carcass	FOX-TET-AXO-AUG2-CIP-NAL-XNL-AMP	*bla*_CMY–2_, *fosA7*, *sul2*, *tet(A), aac(6′)-Iaa, gyrA:p.S83F, parC:p.T57S*	ST15
SSc155 GCA_006209245.1	DF/2016	Slaughterhouse	Chicken carcass	FOX-TET-AXO-AUG2-CIP-NAL-XNL-AMP	*bla*_CMY–2_, *fosA7*, *sul2*, *tet(A), aac(6′)-Iaa, gyrA:p.S83F, parC:p.T57S*	ST15
SH268 GCA_010979095.1	PR/2016	Slaughterhouse	Chicken carcass after chiller	FOX-TET-AXO-AUG2-CIP-NAL-XNL-AMP	*bla*_CMY–2_, *fosA7*, *sul2*, *tet(A), aac(6′)-Iaa, gyrA:p.S83F, parC:p.T57S*	ST15
SH269 GCA_011157135.1	PR/2016	Slaughterhouse	Chicken carcass after chiller	FOX-TET-AXO-AUG2-CIP-NAL-XNL-AMP	*bla*_CMY–2_, *fosA7*, *sul2*, *tet(A), aac(6′)-Iaa, gyrA:p.S83F, parC:p.T57S*	ST15
SH270 GCA_010977655.1	PR/2016	Slaughterhouse	Chicken carcass after chiller	FOX-TET-AXO-AUG2-CIP-NAL-XNL-AMP	*bla*_CMY–2_, *fosA7*, *sul2*, *tet(A), aac(6′)-Iaa, gyrA:p.S83F, parC:p.T57S*	ST15
SH1 GCA_011149295.1	SP/2016	Slaughterhouse	Mechanically recovered chicken meat	FOX-TET-AXO-AUG2-CIP-NAL-XNL-AMP	*bla*_CMY–2_, *fosA7*, *sul2*, *tet(A), aac(6′)-Iaa, gyrA:p.S83F, parC:p.T57S*	ST15
SH131 GCA_006211165.1	SP/2016	Slaughterhouse	Mechanically recovered chicken meat	FOX-TET-AXO-AUG2-CIP-NAL-XNL-AMP	*bla*_CMY–2_, *fosA7*, *sul2*, *tet(A), aac(6′)-Iaa, gyrA:p.S83F, parC:p.T57S*	ST15
SH296 GCA_006292135.1	SP/2016	Slaughterhouse	Mechanically recovered	FOX-TET-AXO-AUG2-CIP-NAL-XNL-AMP-STR	*bla*_CMY–2_, *fosA7*, *sul2*, *tet(A), aac(6′)-Iaa, gyrA:p.S83F, parC:p.T57S*	ST15
SH297 GCA_003877075.1	SP/2016	Slaughterhouse	Mechanically recovered chicken meat	FOX-TET-AXO-AUG2-CIP-NAL-XNL-AMP	*bla*_CMY–2_, *fosA7*, *sul2*, *tet(A), aac(6′)-Iaa, gyrA:p.S83F, parC:p.T57S*	ST15
SH697 GCA_003874475.1	SC/2016	Slaughterhouse	Mechanically recovered chicken meat	FOX-TET-AXO-AUG2-CIP-NAL-XNL-AMP	*bla*_CMY–2_, *fosA7*, *sul2*, *tet(A), aac(6′)-Iaa, gyrA:p.S83F, parC:p.T57S*	ST15
SH700 GCA_006291975.1	SC/2016	Slaughterhouse	Mechanically recovered chicken meat	FOX-TET-AXO-AUG2-CIP-NAL-XNL-AMP	*bla*_CMY–2_, *fosA7*, *sul2*, *tet(A), aac(6′)-Iaa, gyrA:p.S83F, parC:p.T57S*	ST15
SH712 GCA_006210745.1	SP/2016	Slaughterhouse	Mechanically recovered chicken meat	FOX-TET-AXO-AUG2-CIP-NAL-XNL-AMP	*bla*_CMY–2_, *fosA7*, *sul2*, *tet(A), aac(6′)-Iaa, gyrA:p.S83F, parC:p.T57S*	ST15
SH164 GCA_010875785.1						
GCA_010875785.1	SP/2016	Slaughterhouse	Viscera	FOX-TET-AXO-AUG2-CIP-NAL-XNL-AMP	*bla*_CMY–2_, *fosA7*, *sul2*, *tet(A), aac(6′)-Iaa, gyrA:p.S83F, parC:p.T57S*	ST15
SH118 GCA_011163895.1	SP/2016	Retail market	Chicken breast	FOX-TET-AXO-AUG2-CIP-NAL-XNL-AMP	*bla*_CMY–2_ *, fosA7, sul2, tet(A), aac(6′)-Iaa, gyrA:p.S83F, parC:p.T57S*	ST15
SH276 GCA_011571185.1	SP/2016	Retail market	Salted chicken breast	TET-CIP-NAL	*fosA7*, *sul2*, *tet(A), aac(6′)-Iaa, gyrA:p.S83F, parC:p.T57S*	ST15
SH405 GCA_006332425.1	SC/2016	Retail market	Chicken breast fillet	FOX-TET-AXO-AUG2-CIP-NAL-XNL-AMP	*bla*_CMY–2_, *fosA7*, *sul2*, *tet(A), aac(6′)-Iaa, gyrA:p.S83F, parC:p.T57S*	ST15
SH410 GCA_006209405.1	SC/2016	Retail market	Chicken breast fillet	FOX-TET-AXO-AUG2-CIP-NAL-XNL-AMP	*bla*_CMY–2_, *fosA7*, *sul2*, *tet(A), aac(6′)-Iaa, gyrA:p.S83F, parC:p.T57S*	ST15
SH694 GCA_006291675.1						
GCA_006291675.1	SC/2016	Retail market	Chicken breast fillet	FOX-TET-AXO-AUG2-CIP-NAL-XNL-AMP	*bla*_CMY–2_, *fosA7*, *sul2*, *tet(A), aac(6′)-Iaa, gyrA:p.S83F, parC:p.T57S*	ST15
SH120 GCA_011590585.1	SP/2016	Retail market	Chicken thigh and drumstick	FOX-TET-AXO-AUG2-CIP-NAL-XNL-AMP	*bla*_CMY–2_ *, fosA7, sul2, tet(A), aac(6′)-Iaa, gyrA:p.S83F, parC:p.T57S*	ST15
SH286 GCA_006291895.1	SP/2016	Retail market	Chicken thigh and drumstick	FOX-TET-AXO-AUG2-CIP-NAL-XNL-AMP	*bla*_CMY–2_, *fosA7*, *sul2*, *tet(A), aac(6′)-Iaa, gyrA:p.S83F, parC:p.T57S*	ST15
SH411 GCA_006209285.1	SC/2016	Retail market	Chicken thigh and drumstick	FOX-TET-AXO-AUG2-CIP-NAL-XNL-AMP	*bla*_CMY–2_, *fosA7*, *sul2*, *tet(A), aac(6′)-Iaa, gyrA:p.S83F, parC:p.T57S*	ST15
SH692 GCA_006211665.1	SC/2016	Retail market	Chicken thigh and drumstick	FOX-TET-AXO-AUG2-CIP-NAL-XNL-AMP	*bla*_CMY–2_, *fosA7*, *sul2*, *tet(A), aac(6′)-Iaa, gyrA:p.S83F, parC:p.T57S*	ST15
SH121 GCA_010946195.1	SP/2016	Retail market	Chicken fillet sassami	FOX-TET-AXO-AUG2-CIP-NAL-XNL-AMP	*bla*_CMY–2_ *, fosA7, sul2, tet(A), aac(6′)-Iaa, Inu(G), gyrA:p.S83F, parC:p.T57S*	ST15
SH127 GCA_011146395.1	SP/2016	Retail market	Chicken fillet sassami	FOX-TET-AXO-AUG2-CIP-NAL-XNL-AMP	*bla*_CMY–2_ *, fosA7, sul2, tet(A), aac(6′)-Iaa, Inu(G), gyrA:p.S83F, parC:p.T57S*	ST15
SH135 GCA_011146615.1	SP/2016	Retail market	Whole chicken	FOX-TET-AXO-AUG2-CIP-NAL-XNL-AMP-STR	*bla*_CMY–2_, *fosA7*, *sul2*, *tet(A), aac(6′)-Iaa, gyrA:p.S83F, parC:p.T57S*	ST15
SH427 GCA_003877155.1	SC/2016	Retail market	Whole chicken	FOX-TET-AXO-AUG2-CIP-NAL-XNL-AMP-STR	*bla*_CMY–2_, *fosA7*, *sul2*, *tet(A), aac(6′)-Iaa, gyrA:p.S83F, parC:p.T57S*	ST15
SH138 GCA_010980075.1	SC/2016	Retail market	Leg quarter	FOX-TET-AXO-AUG2-CIP-NAL-XNL-AMP-STR	*bla*_CMY–2_, *fosA7*, *sul2*, *tet(A), aac(6′)-Iaa, gyrA:p.S83F, parC:p.T57S*	ST15
SH158 GCA_010902135.1	MG/2016	Retail market	Fiesta boneless	FOX-TET-AXO-AUG2-CIP-NAL-XNL-AMP-STR	*bla*_CMY–2_ *, fosA7, aadA1, aadA2, aac(6′)-Iaa, cmlA1, dfrA12, sul2, sul3, tet(A), qacL, gyrA:p.S83F, parC:p.T57S*	ST15
SH287 GCA_004158845.1	SP/2016	Retail market	Chicken skin	FOX-TET-AXO-AUG2-CIP-NAL-XNL-AMP-STR	*bla*_CMY–2_, *fosA7*, *sul2*, *tet(A), aac(6′)-Iaa, gyrA:p.S83F, parC:p.T57S*	ST15
SH289 GCA_004159315.1	SP/2016	Retail market	Seasoned chicken fillet	FOX-TET-AXO-AUG2-CIP-NAL-XNL-AMP	*bla*_CMY–2_, *fosA7*, *sul2*, *tet(A), aac(6′)-Iaa, gyrA:p.S83F, parC:p.T57S*	ST15
SH403 GCA_006292115.1	SC/2016	Retail market	Seasoned chicken fillet	FOX-TET-AXO-AUG2-CIP-NAL-XNL-AMP	*bla*_CMY–2_, *fosA7*, *sul2*, *tet(A), aac(6′)-Iaa, gyrA:p.S83F, parC:p.T57S*	ST15
SH290 GCA_006332625.1	SP/2016	Retail market	Chicken liver	FOX-TET-AXO-AUG2-CIP-NAL-XNL-AMP	*bla*_CMY–2_, *fosA7*, *sul2*, *tet(A), aac(6′)-Iaa, gyrA:p.S83F, parC:p.T57S*	ST15
SH402 GCA_006332585.1	SC/2016	Retail market	Chicken liver	FOX-TET-AXO-AUG2-CIP-NAL-XNL-AMP	*bla*_CMY–2_, *fosA7*, *sul2*, *tet(A), aac(6′)-Iaa, gyrA:p.S83F,parC:p.T57S*	ST15
SH408 GCA_006291855.1	SC/2016	Retail market	Chicken liver	FOX-TET-AXO-AUG2-CIP-NAL-XNL-AMP	*bla*_CMY–2_, *fosA7*, *sul2*, *tet(A), aac(6′)-Iaa, gyrA:p.S83F, parC:p.T57S*	ST15
SH422 GCA_006291955.1	SC/2016	Retail market	Chicken liver	FOX-TET-AXO-AUG2-CIP-NAL-XNL-AMP	*bla*_CMY–2_, *fosA7*, *sul2*, *tet(A), aac(6′)-Iaa, gyrA:p.S83F, parC:p.T57S*	ST15
SH423 GCA_006209445.1	SC/2016	Retail market	Chicken liver	FOX-TET-AXO-AUG2-CIP-NAL-XNL-AMP	*bla*_CMY–2_, *fosA7*, *sul2*, *tet(A), aac(6′)-Iaa, gyrA:p.S83F, parC:p.T57S*	ST15
SH429 GCA_004160665.1	SC/2016	Retail market	Chicken liver	FOX-TET-AXO-AUG2-CIP-NAL-XNL-AMP	*bla*_CMY–2_, *fosA7*, *sul2*, *tet(A), aac(6′)-Iaa, gyrA:p.S83F, parC:p.T57S*	ST15
SH430 GCA_006291835.1	SC/2016	Retail market	Chicken liver	FOX-TET-AXO-AUG2-CIP-NAL-XNL-AMP	*bla*_CMY–2_, *fosA7*, *sul2*, *tet(A), aac(6′)-Iaa, gyrA:p.S83F, parC:p.T57S*	ST15
SH431 GCA_006210515.1	SC/2016	Retail market	Chicken liver	FOX-TET-AXO-AUG2-CIP-NAL-XNL-AMP	*bla*_CMY–2_, *fosA7*, *sul2*, *tet(A), aac(6′)-Iaa, gyrA:p.S83F, parC:p.T57S*	ST15
SH674 GCA_006332645.1	SC/2016	Retail market	Chicken liver	FOX-TET-AXO-AUG2-CIP-NAL-XNL-AMP	*bla*_CMY–2_, *fosA7*, *sul2*, *tet(A), aac(6′)-Iaa, gyrA:p.S83F, parC:p.T57S*	ST15
SH687 GCA_006211605.1	SC/2016	Retail market	Chicken liver	FOX-TET-AXO-AUG2-CIP-NAL-XNL-AMP	*bla*_CMY–2_, *fosA7*, *sul2*, *tet(A), aac(6′)-Iaa, gyrA:p.S83F, parC:p.T57S*	ST15
SH707 GCA_006211425.1	SC/2016	Retail market	Chicken liver	FOX-TET-AXO-AUG2-CIP-NAL-XNL-AMP	*bla*_CMY–2_, *fosA7*, *sul2*, *tet(A), aac(6′)-Iaa, gyrA:p.S83F, parC:p.T57S*	ST15
SH412 GCA_004159355.1	SC/2016	Retail market	Chicken wing	FOX-TET-AXO-AUG2-CIP-NAL-XNL-AMP	*bla*_CMY–2_, *fosA7*, *sul2*, *tet(A), aac(6′)-Iaa, gyrA:p.S83F, parC:p.T57S*	ST15
SH680 GCA_003877135.1	SC/2016	Retail market	Retail meat	FOX-TET-AXO-AUG2-CIP-NAL-XNL-AMP	*bla*_CMY–2_, *fosA7*, *sul2*, *tet(A), aac(6′)-Iaa, gyrA:p.S83F, parC:p.T57S*	ST15
SH681 GCA_006292015.1	SC/2016	Retail market	Chicken wing	FOX-TET-AXO-AUG2-CIP-NAL-XNL-AMP	*bla*_CMY–2_, *fosA7*, *sul2*, *tet(A), aac(6′)-Iaa, gyrA:p.S83F, parC:p.T57S*	ST15
SH685 GCA_004161515.1	SC/2016	Retail market	Chicken neck	FOX-TET-AXO-AUG2-CIP-NAL-XNL-AMP-STR	*bla*_CMY–2_, *fosA7*, *sul2*, *tet(A), aac(6′)-Iaa, gyrA:p.S83F, parC:p.T57S*	ST15
SH691 GCA_006291915.1	SC/2016	Retail market	Chicken wing	FOX-TET-AXO-AUG2-CIP-NAL-XNL-AMP-STR	*bla*_CMY–2_, *fosA7*, *sul2*, *tet(A), aac(6′)-Iaa, gyrA:p.S83F, parC:p.T57S*	ST15
SH693 GCA_006210725.1	SC/2016	Retail market	Chicken wing	FOX-TET-AXO-AUG2-CIP-NAL-XNL-AMP	*bla*_CMY–2_, *fosA7*, *sul2*, *tet(A), aac(6′)-Iaa, gyrA:p.S83F, parC:p.T57S*	ST15
SSc139 GCA_011578645.1	SP/2016	Retail market	Chicken wing	FOX-TET-AXO-AUG2-CIP-NAL-XNL-AMP-STR	*bla*_CMY–2_, *fosA7*, *sul2*, *tet(A), aac(6′)-Iaa, gyrA:p.S83F, parC:p.T57S*	ST15
SH716 GCA_006332605.1	SC/2016	Retail market	Chicken wing paddle	FOX-TET-AXO-AUG2-CIP-NAL-XNL-AMP	*bla*_CMY–2_, *fosA7*, *sul2*, *tet(A), aac(6′)-Iaa, gyrA:p.S83F, parC:p.T57S*	ST15
SSC136 GCA_010932755.1	SP/2016	Retail market	Chicken wing	FOX-TET-AXO-AUG2-CIP-NAL-XNL-AMP	*bla*_CMY–2_, *fosA7*, *sul2*, *tet(A), aac(6′)-Iaa, gyrA:p.S83F, parC:p.T57S*	ST15

Minimum inhibitory concentrations (MICs) were determined by broth microdilution using Sensititre^®^ Gram-Negative Plates (Trek Diagnostic Systems, OH), such as 14 antimicrobials: cefoxitin (FOX), ceftriaxone (AXO), amoxicillin/clavulanic acid 2:1 ratio (AUG2), ceftiofur (XNL), ampicillin (AMP), nalidixic acid (NAL), ciprofloxacin (CIP), chloramphenicol (CHL), tetracycline (TET), gentamicin (GEN), sulfisoxazole (FIS), trimethoprim/sulfamethoxazole (SXT), streptomycin (STR), and azithromycin (AZI). MIC values were interpreted according to the guidelines of the Clinical and Laboratory Standards Institute (CLSI) (Clinical and Laboratory Standards Institute [CLSI], 2021) and the National Antimicrobial Resistance Monitoring System (US Food and Drug Administration [FDA], 2015). Multidrug resistance was defined as resistant to three or more classes of antimicrobials (Magiorakos et al., 2012).

### Genomic Analysis

All *S*. Heidelberg isolates (*n* = 81) underwent DNA extraction performed by using a commercial kit (QiAmp tissue, Qiagen, Germany) per manufacturer’s guidelines. Genomic DNA of eighty-one *Salmonella* isolates was sequenced at a 300-bp paired-end-read using the Nextera XT library preparation kit at the MiSeq platform (Illumina, San Diego, CA, United States).

Resulted raw sequence reads underwent strict quality control by using default settings in CLC workbench 10.1.1 (Qiagen) as per Monte et al. (2019), while assemblies were annotated with PROKKA version 1.14-dev (Seemann, 2014). A core genome phylogeny was constructed with an alignment of the core genes determined by the software version 3.11.2; the BlastP threshold was set to 95% (Page et al., 2015). A pan-genome genes presence–absence information from Roary was visualized with Phandango (Hadfield et al., 2018). The single nucleotide polymorphisms were extracted from the alignment using SNP-sites version 2.3.3 (Page et al., 2016). The phylogeny was reconstructed using RAxML version 8.2.12, using a General Time Reversible Model and Gamma distribution for rate heterogeneity (Stamatakis, 2014). The resulting phylogeny was tested against 1,000 bootstrap replications, as determined by implementing the majority rule, autoMR convergence criteria in the RAxML software (Pattengale et al., 2010). The phylogeny was visualized and annotated using iTol version 3 (Letunic and Bork, 2016).

Lastly, the assemblies were analyzed for acquired AMR genes and chromosomal point mutations using default settings of ResFinder 4.1 database available at the Center for Genome Epidemiology.^1^ In addition, we used MLST 2.0 to detect multilocus sequence typing (MLST), and the PlasmidFinder software version 2.0.1 was run with database version 2018-11-20 (Carattoli et al., 2014). A minimum identity threshold of 95% was used as a filter for identification.

### Clustered Regularly Interspaced Short Palindromic Repeats Genotyping and Phylogenetic Analysis

An automated high-throughput processing pipeline previously described by Nethery and Barrangou (2019) was used to identify the CRISPR loci within each strain. Using CRISPR Visualizer, we extracted and imported CRISPR loci into the web interface for visualization and alignment of all CRISPR spacer and repeat sequences.^2^

## Results

### *Salmonella* Heidelberg Strains Harbored a Wide Resistome Against Critically Important Antimicrobials

A total of 81 (100%) *S*. Heidelberg strains were both phenotypically and genotypically resistant, whereas 91.3% (*n* = 74) were multidrug-resistant, defined as resistant to three or more classes of antimicrobial compounds (Magiorakos et al., 2012; Table 1). Results of the antimicrobial susceptibility testing are presented in Table 2. MICs vary among *S.* Heidelberg strains. All *S.* Heidelberg strains were resistant to TET, NAL, CIP, and FIS with MIC values ranging from 0.25 to ≥ 256 μg/ml (Table 2). The high MIC values observed in this study for β-lactams (AMP, amoxicillin/clavulanic acid, AXO, XNL, and FOX), TET, FIS, NAL, and STR (Table 2), confirm the high frequency of AMR genes and mutations predicted by genomic analysis. Yet, based on the MIC distribution, all *S*. Heidelberg strains displayed susceptibility to AZI (Table 2).

**TABLE 2:** Minimum inhibitory concentration values for *Salmonella* Heidelberg strains (*n* = 81).

Antimicrobials	Resistance (%)	Intermediate resistance (%)	Distribution of *S.* Heidelberg strains (*n* = 81) among MIC values (μ g/ml)*^a^*															
			
			0.015	0.03	0.06	0.12	0.25	0.5	1	2	4	8	16	32	64	128	256	≥512
Cefoxitin	88.8	0								6	2	1		**72**				
Azithromycin	12.3	0									22	49	10					
Chloramphenicol	1.23	12.3									7	63	10	**1**				
Tetracycline	100	0												**81**				
Ceftriaxone	88.8	0	9									**2**	**45**	**20**	**5**			
Amoxicillin/clavulanic acid	88.8	0	6							3				**72**				
Ciprofloxacin	100	0					**35**	**36**	**10**									
Gentamicin	1.23	0						64	16				**1**					
Nalidixic acid	100	0												**81**				
Ceftiofur	88.8	0	1						8			**72**						
Sulfisoxazole	100	0															**81**	
Trimethoprim/sulfamethoxazole	1.23	0	72				7	1			**1**							
Ampicillin	88.8	0	2						1	5	1			**72**				
Streptomycin	16.0	0											68	**11**	**2**			

*^a^Blue MIC values indicate intermediate resistance, while red MIC values in gray squares indicate resistance profiles, which were determined by broth microdilution method using CLSI interpretative breakpoints (Clinical and Laboratory Standards Institute [CLSI], 2021).*

**FIGURE 1:**
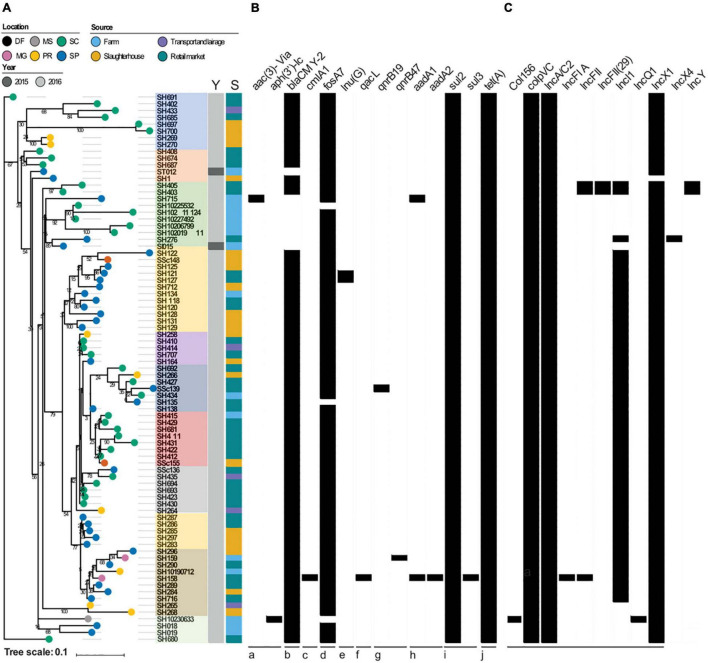
**(A)** Reconstructed phylogeny based on the core genome (4,139 genes) of the 81 *S*. Heidelberg strains. The percentage of bootstrap samples in which nodes appeared is shown. The location of isolation of each strain is labeled on its respective branch. Color strips depict the year (Y) and source (S) of isolation, respectively. **(B)** The presence and absence of selected antimicrobial resistance genes are shown, with black indicating presence. The drug classes impacted by these genes are: (a) aminoglycosides, (b) beta-lactams, (c) chloramphenicol, (d) fosfomycin, (e) lincosamide, (f) quaternary ammonium compounds, (g) quinolones, (h) streptomycin, (i) sulfonamides, (j) tetracylines. **(C)** Presence and absence of plasmid incompatibility groups, with black indicating presence. Brazilian States: PR, Paraná; SC, Santa Catarina; SP, São Paulo; MG, Minas Gerais; DF, Distrito Federal; MS, Mato Grosso do Sul.

The total AMR gene abundances did not vary significantly across regions and sources suggesting pervasive distribution of antibiotic resistant strains from farm to market in six different States of Brazil (Figure 1). The highest AMR gene abundances were observed for fosfomycin (*fosA7*; 100%), sulfonamide (*sul2*; 100%), tetracycline [*tet(A)*; 100%], and aminoglycoside [*aac(6′)-Iaa*; 100%]. Seventy-two (88.8%) *S*. Heidelberg strains harbored the plasmid-mediated AmpC β-lactamase (*bla*_*CMY*–2_), encoding resistance to third-generation cephalosporin (3GC). Unlike, *Inu(G)* (*n* = 2), *aadA1* (*n* = 2), *aph(3′)-Ia* (*n* = 1), *aac(3)-Via* (*n* = 1), *aadA2* (*n* = 1), *cmlA1* (*n* = 1), *dfrA12* (*n* = 1), *sul3* (*n* = 1), and *qacL* (*n* = 1) AMR genes were detected at very low levels (Table 1). On the other hand, chromosomal point mutations in *gyrA* [p. Ser83Phe (tcc → ttc)] and *parC* [p. Thr57Ser (acc → agc)] were identified in 100% of the strains. This quinolone resistance-determining region (QRDR) among *S*. Heidelberg strains was sufficient to promote high-level resistance at > 32 μg/ml for NAL.

All 81 *Salmonella* genomes were analyzed for the content of plasmid replicons by using the Center for Genomic Epidemiology (CGE) web-tool PlasmidFinder 2.1, with 100% of the genomes containing at least two replicons, like ColpVC and IncA/C2. The remaining plasmids replicons such as IncX1 (*n* = 80; 98.7%), Incl1 (*n* = 56; 69.1%), IncFII (*n* = 3; 3.7%), IncFII(29) (*n* = 2; 2.4%), IncY (*n* = 2; 2.4%), Col156 (*n* = 1; 1.2%), IncFIA (*n* = 1; 1.2%), IncQ1(*n* = 1; 1.2%), and IncX4 (*n* = 1; 1.2%) were identified within *S*. Heidelberg genomes (Figure 1).

### Spacer Composition and Sequence Type Were Highly Conserved Within *Salmonella* Heidelberg Strains

**FIGURE 2:**
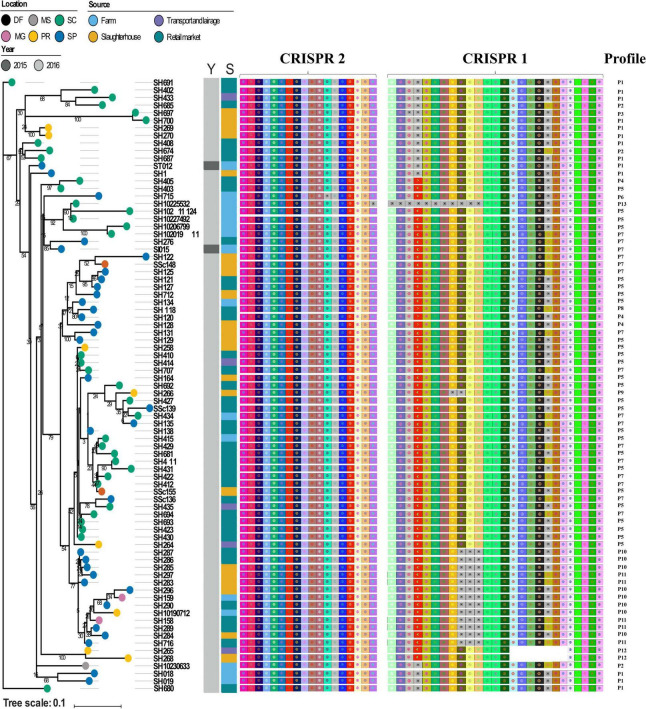
Reconstructed phylogeny based on the core genome, distribution of spacers composition, clustered regularly interspaced short palindromic repeats (CRISPR) loci, and CRISPR profiles among *Salmonella* Heidelberg strains. The location of isolation of each strain is labeled on its respective branch. Color strips depict the year (Y) and source (S) of isolation, respectively. Brazilian States: PR, Paraná; SC, Santa Catarina; SP, São Paulo; MG, Minas Gerais; DF, Distrito Federal; MS, Mato Grosso do Sul.

**FIGURE 3:**
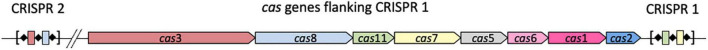
*Salmonella enterica* subsp. *enterica* serovar Heidelberg type I-E CRISPR locus architecture. This system contains two distinct CRISPR arrays—one associated with the *cas* genes and one disparate locus upstream (5′) from the *cas* genes.

Next, we visualized CRISPR loci extracted from WGS data to analyze the pattern of repeats and spacers distributed among *S*. Heidelberg strains (*n* = 81). In doing so, we observed 13 unique CRISPR array patterns [P1 (*n* = 13), P2 (*n* = 3), P3 (*n* = 1), P4 (*n* = 3), P5 (*n* = 26), P6 (*n* = 1), P7 (*n* = 16), P8 (*n* = 1), P9 (*n* = 1), P10 (*n* = 8), P11 (*n* = 5), P12 (*n* = 2), and P13 (*n* = 1)] as shown in Table 3. Overall, we observed a maximum of 44 spacers across *S*. Heidelberg strains (P7), spread across two loci. SH265 and SH268, belonging to profile P12, contained 37 spacers, the lowest number presented here. Spacer composition was highly conserved across strains, which shared 43 (P1, P2, P3, P4, P5, and P6), 42 (P7 and P8), 40 (P9 and P10), 39 (P11), and 36 (P12) identical spacers, reflecting a common ancestral origin (Figure 2). Next, we performed a comparative analysis of the architecture of the type I-E CRISPR-Cas system present in these strains and observed 100% amino acid identity across all strains—further evidence of shared ancestral origin (Figure 3). We further evaluated the multi-locus sequence typing by *in silico* prediction, which revealed that all *S*. Heidelberg strains matched the international sequence type (ST15) (Table 1).

**TABLE 3:** Clustered regularly interspaced short palindromic repeats (CRISPR) patterns obtained from 81 *Salmonella* Heidelberg strains.

CRISPR profile	Location*	Source	Year of isolation
P1	SC (*n* = 7), PR (*n* = 2), SP (*n* = 4)	Retail market (*n* = 5), transport and lairage (*n* = 1), slaughterhouse (*n* = 4), broiler farm (*n* = 3)	2015 (*n* = 1), 2016 (*n* = 12)
P2	SC (*n* = 2), MS (*n* = 1)	Retail market (*n* = 2), broiler farm (*n* = 1)	2016 (*n* = 3)
P3	SC (*n* = 1)	Slaughterhouse (*n* = 1)	2016 (*n* = 1)
P4	SC (*n* = 1), SP (*n* = 2)	Retail market (*n* = 2), slaughterhouse (*n* = 1)	2016 (*n* = 3)
P5	SC (*n* = 15), SP (*n* = 8), PR (*n* = 2), DF (*n* = 1)	Retail market (*n* = 13), transport and lairage (*n* = 3), slaughterhouse (*n* = 5), broiler farm (*n* = 5)	2016 (*n* = 26)
P6	SP (*n* = 1)	Broiler farm (*n* = 1)	2016 (*n* = 1)
P7	SC (*n* = 8), SP (*n* = 7), DF (*n* = 1)	Retail market (*n* = 9), slaughterhouse (*n* = 4), broiler farm (*n* = 3)	2015 (*n* = 1), 2016 (*n* = 15)
P8	SP (*n* = 1)	Retail market (*n* = 1)	2016 (*n* = 1)
P9	PR (*n* = 1)	Slaughterhouse (*n* = 1)	2016 (*n* = 1)
P10	SP (*n* = 6), MG (*n* = 1), PR (*n* = 1)	Retail market (*n* = 3), slaughterhouse (*n* = 3), broiler farm (*n* = 2)	2016 (*n* = 8)
P11	SP (*n* = 4), MG (*n* = 1)	Retail market (*n* = 3), slaughterhouse (*n* = 2)	2016 (*n* = 5)
P12	PR (*n* = 2)	Transport and lairage (*n* = 1), slaughterhouse (*n* = 1)	2016 (*n* = 2)
P13	SC (*n* = 1)	Broiler farm (*n* = 1)	2016 (*n* = 1)

*Brazilian States: PR, Paraná; SC, Santa Catarina; SP, São Paulo; MG, Minas Gerais; DF, Distrito Federal; MS, Mato Grosso do Sul.

### *Salmonella* Heidelberg Strains Isolated From Brazil Are Genetically Related to South American, European, and Asian Isolates

While assessing the phylogenetics of *S*. Heidelberg strains (*n* = 81) sequenced in this study, we noticed that the core genome, calculated from WGS data, represented 74% of the pan-genome (4,139 out of 5,582 total genes). Little genomic variation was present among the core genome, as only 704 SNP sites were detected. Bootstrap values varied across the phylogeny, likely attributed to the small genomic variation among strains. *S*. Heidelberg strains did not cluster by year, source, or geographic location across the phylogeny suggesting the widespread distribution [regions (*n* = 6), sources (*n* = 4), years of isolation (*n* = 2)] and persistence of *Salmonella* strains in Brazil (Figure 1), which validate the previous surveys (Monte et al., 2019). Furthermore, 11 different clusters were identified as shown in Figure 1. Of these, 14 strains appear to be from independent lineages, given that they nested out of the main branches.

We also observed through SNP clustering (PDS000037185.127; *n* = 765 isolates; NCBI pathogen detection tool), cases of international clustering of *S*. Heidelberg from our collection (*n* = 77) with strains isolated from a variety of sources (food, human, and environment) from Brazil (*n* = 201), Chile (*n* = 9), the United Kingdom (*n* = 444), Germany (*n* = 1), the Netherlands (*n* = 2), South Korea (*n* = 1), and China (*n* = 1), which suggest a common ancestor origin (Supplementary Figure 1). Another two strains (SH265 and SH268) from this study nested in the SNP cluster (PDS000029160.10) with strains isolated from Brazil (*n* = 11), the United Kingdom (*n* = 4), and Chile (*n* = 1) (Supplementary Figure 2).

## Discussion

There has been a great interest in surveying the adaptation of *Salmonella* serovars to the poultry production chain because of their extensive persistence in the past, notably with *S*. Typhimurium and *S*. Enteritidis, which have caused significant economic losses to this sector. Furthermore, the prevalence of *S*. Heidelberg shown in this study is not the only issue, but the fact that highly drug-resistant and/or MDR isolates are being recovered in most steps of the poultry production chain, particularly in Brazil could be considered a public health threat, as there is a risk of it becoming globalized.

Based on AMR results, the β-lactam resistance was essentially driven by the presence of plasmid-mediated AmpC (pAmpC) *bla*_*CMY*–2_ gene, given the isolates which did not carry this gene were susceptible to FOX, while QRDR such as *gyrA* and *parC* genes drove quinolone resistance (Table 1). Indeed, the presence of strains harboring *bla*_*CMY*–2_ gene could have implications on a one health interface, since this plasmid is more likely to persist (Teunis et al., 2018). Besides that, all strains harbored chromosomal mutations in *gyrA* and *parC* genes promoting high-level resistance against quinolones that could have implications on human health as treatment options become limited. Disturbingly, this result corroborates the findings by van den Berg et al. (2019) that found 98.4% of the *S*. Heidelberg isolates resistant to fluoroquinolones. On the other hand, all *S*. Heidelberg strains from our collection, displayed susceptibility to azithromycin, which could be considered a promising agent against Salmonellae infections (Crump et al., 2015; Wen et al., 2017). Azithromycin has been used as an alternative treatment option for enteric fever even when the guidelines on susceptibility testing were not available. Like fluoroquinolones, azithromycin is an antimicrobial agent with efficient intracellular penetration (Crump et al., 2015; Wen et al., 2017).

The total AMR load also included encoding resistance genes for aminoglycoside [*aac(3)-VIa*, *aph(3′)-Ic, aadA1, aadA2*], chloramphenicol (*cmlA1*), macrolides [*Inu(G)*], trimethoprim (*dfrA12*), and ammonium quaternary compounds (*qacL*) (Figure 1). Interestingly, the unique strain (SSc139) that carried the *qacL* gene was isolated from the retail market, which makes large use of such compounds to disinfect surfaces. Moreover, this strain nested in the same cluster with five strains isolated from broiler farms and slaughterhouse sources, in different states such as São Paulo, Santa Catarina, and Parana, denoting the successful establishment of this lineage (ST15) in the Southern and South regions of Brazil. In this regard, we visualized the co-occurrence of AMR genes in all *S*. Heidelberg strains.

The presence of AMR genes and transmissible plasmids demonstrated little variation across the strains. The broad distribution and abundance of *S*. Heidelberg in broiler farms, slaughterhouses, transport, lairages, and retail markets suggest the high adaptability of this serovar in the poultry production chain in Brazil. Similarly, a study by Edirmanasinghe et al. (2017) examining FOX-resistant *S*. Heidelberg strains isolated from human, abattoir poultry, and retail poultry sources in Canada revealed a potential common source among strains, which suggest the simultaneous dispersal of *S*. Heidelberg strains carrying CMY-2 gene in several sources and different geographical locations. In convergence with our results, another study revealed a high occurrence of *S*. Heidelberg in imported poultry meat in the Netherlands containing *bla*_*CMY*–2_ gene (van den Berg et al., 2019). Although we could not find colistin-resistant strains, it is worthwhile to note the presence of the IncX4 plasmid since it is a promiscuous plasmid with a high capacity of self-transmissibility that is commonly associated with the presence of plasmid-mediated colistin-resistance (*mcr-1*) in Brazil (Moreno et al., 2019).

Consistent with observations obtained in our study, recent surveys strongly support that *S*. Heidelberg may originate from a common ancestor, which circulates and persists in the Brazilian poultry production chain since at least 2004 (Kipper et al., 2021). More importantly, these strains are also nested with strains isolated from several sources and countries around the world supporting the hypotheses of intercontinental spread, which demonstrate that probably the common ancestor underwent diversification through genetic changes over time (Supplementary Material).

Protracted dissemination of *S*. Heidelberg *via* poultry might be a risk for a globalized food trade era. The introduction and clonal expansion of *Salmonella* strains across borders remain challenging due to the difficulties of identifying the origins of contamination. In light of this, the continued need for combined approaches between classical microbiology and high-resolution methods such as WGS and CRISPR genotyping truly illustrate to us what is hidden in plain sight.

For the purpose of discussion, other studies provide compelling validation data to support the usefulness of high-resolution methods for genotyping rare *Salmonella enterica* serovars (Monte et al., 2021) and/or to resolve *S*. Heidelberg isolates involved in foodborne outbreaks (Vincent et al., 2018). Of the latter, while assessing the CRISPR array of 145 *S*. Heidelberg isolates, Vincent et al. (2018) found 15 different CRISPR profiles endorsing our results.

This study further illustrates the potential of CRISPR for the tracking of variable genotypes in diverse *Salmonella* strains, as previously determined (DiMarzio et al., 2013; Shariat et al., 2013a,b, 2015; Monte et al., 2021), with noteworthy methodological convenience. Indeed, CRISPR-based analyses have proven relevant for subtyping of *Salmonella enterica* serovars Typhimurium and Heidelberg strains involved in outbreaks (Shariat et al., 2013b) and occasionally associated with antibiotic resistance (DiMarzio et al., 2013). Our findings underscore the potential role of *S*. Heidelberg as a key pathogen in the poultry production chain, particularly in Brazil.

## Data Availability Statement

The datasets presented in this study can be found in online repositories. The names of the repository/repositories and accession number(s) can be found in the article/Supplementary Material.

## Author Contributions

DM, RB, MN, PF-C, and ML designed the study. RB, PF-C, NL, and ML supervised the work. DM, MN, HB, SK, NL, PF-C, RB, and ML participated, coordinated, and analyzed the data. DM, MN, and RB wrote the original draft. All authors approved the final manuscript.

## Conflict of Interest

The authors declare that the research was conducted in the absence of any commercial or financial relationships that could be construed as a potential conflict of interest.

## Publisher’s Note

All claims expressed in this article are solely those of the authors and do not necessarily represent those of their affiliated organizations, or those of the publisher, the editors and the reviewers. Any product that may be evaluated in this article, or claim that may be made by its manufacturer, is not guaranteed or endorsed by the publisher.

## Footnotes

1
http://www.genomicepidemiology.org/

2
https://github.com/CRISPRlab/CRISPRviz

## Supplementary Material

Supplementary Figures 1, 2 SNP-based phylogram of S. Heidelberg isolates from various isolation sources and locations. Strain ID, isolate source, location, SNP cluster, and collection date were retrieved from Genbank. Red color indicates isolates from this study.
