# Supplementary figures and images for: Clustered Regularly Interspaced Short Palindromic Repeats Genotyping of Multidrug-Resistant Salmonella Heidelberg Strains Isolated From the Poultry Production Chain Across Brazil
Source: Front Microbiol. 2022 Jun 17;13:867278. doi: 10.3389/fmicb.2022.867278 (PMC9248969; doi:10.3389/fmicb.2022.867278)

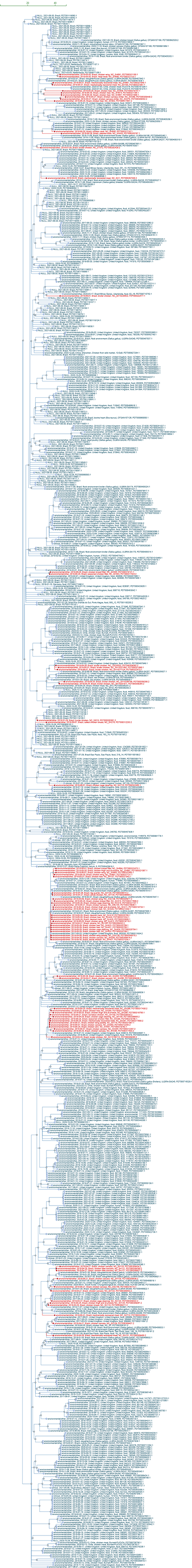

Supplement: Supplementary file 1 [file Image_2.TIFF]

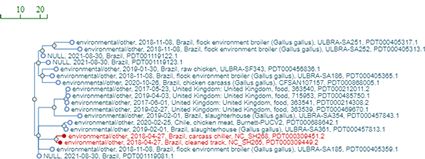

Supplement: Supplementary Figures 1, 2 — SNP-based phylogram of S. Heidelberg isolates from various isolation sources and locations. Strain ID, isolate source, location, SNP cluster, and collection date were retrieved from Genbank. Red color indicates isolates from this study. [file Image_1.TIFF]
